# IVIG Effects on Erythrocyte Sedimentation Rate in Children

**DOI:** 10.1155/2014/981465

**Published:** 2014-01-29

**Authors:** Farhad Salehzadeh, Ahmadvand Noshin, Sepideh Jahangiri

**Affiliations:** Pediatric Department, Bouali Hospital, Ardabil University of Medical Sciences (ARUMS), Ardabil 56157, Iran

## Abstract

*Background*. Erythrocyte sedimentation rate (ESR) is a valuable laboratory tool in evaluation of infectious, inflammatory, and malignant diseases. Red blood cells in outside from the body precipitate due to their higher density than the plasma. In this study we discuss the IVIG effect on ESR in different diseases and different ages. *Methods and Materials*. Fifty patients under 12 years old who had indication to receive IVIG enrolled in this study. Total dose of IVIG was 2 gr/kg (400 mg/kg in five days or 2 gr/kg in single dose). ESR before infusion of IVIG and within 24 hours after administration of the last dose of IVIG was checked. *Results*. 23 (46%) patients were males and 27 (54%) were females. The mean of ESR before IVIG was 31.8 ± 29.04 and after IVIG it was 47.2 ± 36.9; this difference was meaningful (*P* = 0.05). Results of ESR changes in different age groups, 6 patients less than 28 days, 13 patients from 1 month to 1 year, 20 patients from 1 to 6 years old, and 11 patients from 6 to 12 years have been meaningful (*P* = 0.001, *P* = 0.025, and *P* = 0.006, resp.). *Conclusion*. In patients who are receiving IVIG as a therapy, ESR increased falsely (noninflammatory rising); therefore use of ESR for monitoring of response to treatment may be unreliable. Although these results do not apply to neonatal group, we suggest that, in patients who received IVIG, interpretation of ESR should be used cautiously on followup.

## 1. Introduction

The erythrocyte sedimentation rate (ESR) is an acute phase reactant (APR). The rate of sedimentation in a period of one hour called ESR and also Biernacki test. ESR test is a common hematologic nonspecific indicator of inflammation. To perform a test, nonclothing blood is placed in a vertical tube (Westergren) and erythrocyte sedimentation rate is measured and is reported in units of mm/h [1].

The best way to test was presented in 1921 by Westergren, and it is still the golden standard method for measuring erythrocyte sedimentation rate. This method is considered to be [2] simple and cheap, accessible, and accurate [3]. ESR is a valuable laboratory tool in evaluation of infectious, inflammatory, and malignant diseases [1, 3]. Red blood cells in outside from the body precipitate due to their higher density than the plasma; in normal state these cells reject each other because of their negative surface charges and prevent Rolex formation. In order to overcome the negative charge of the red cells should be much stronger gravity. It is exerted by different types of plasma proteins [4].

It was shown that several factors such as PH levels of plasma other than the size of molecules or Rolex formation contribute to erythrocyte sedimentation [2]. ESR levels increase with age and are higher in women [1, 4, 5], anemia, and the black people. Clinical factors that do not influence the ESR are [1, 4] obesity, body temperature, recent food, and NSAID [1, 4, 5].

IVIG with the half-life of 3-4 weeks was first produced in 1960 [6, 7]; IVIG in high doses is used for the treatment of many autoimmune diseases including autoimmune thrombocytopenia, chronic inflammatory polyneuropathy, Kawasaki disease, and Guillain-Barré syndrome [7–9].

High-dose IVIG effects on various proteins, including inflammatory profiles in 63 children with Kawasaki disease, were studied. All children had clinical manifestations of Kawasaki and received 2 gr/kg IVIG and aspirin during 12 hr. serial testing was carried out before receiving IVIG, 24 hours and 7 days later.

After IVIG infusion, total WBC and neutrophils were decreased, whereas lymphocytes were increased. Mean ESR was 12.7 ± 6.46 mm/h before receiving IVIG, 53.3 ± 11.9 mm/h in 24 h, and 48.8 ± 15.2 mm/h in the 7 days after the IVIG infusion. Protein levels which are associated with systemic inflammation except ESR decreased after 24 hours.

IgA and IgM immunoglobulin levels did not change 24 hours and 7 days after injection; however, IgG is significantly increased. The result is that the high dose of IVIG leads to rapid decreased changes of various proteins except for IgA and IgM and ESR [10].

In the same study on patients with myasthenia gravis and Guillain-Barré syndrome, similar results were obtained [11]. In this study we discuss the IVIG effect on ESR in different diseases and ages. We try to answer this question too, is ESR a valuable APR marker in evaluation of inflammatory response to treatment when IVIG is used previously?

## 2. Method and Materials

This is an analytical descriptive and cross-sectional study.

Fifty patients who had indication to receive IVIG enrolled in this study. Total dose of IVIG was 2 gr/kg (400 mg/kg in five days or 2 gr/kg in single dose). ESR before infusion of IVIG and within 24 hours after administration of the last dose of IVIG was checked (1-2 gr/kg/12 h or 400 mg/kg dose IVIG). Brand name for the IVIG was OCTAPHARMA AG, Lachen, Switzerland.

The erythrocyte sedimentation rate was measured by Westergren method; IVIG side effects were not observed in any patient. Results have been shown in different age groups, neonatal, infancy, childhood, and school age.

SPSS 16 and paired *t*-test were used to statistical software analysis; significance level of less than 0.05 was considered meaningful. Consent confirmed was obtained from parents of patients.

## 3. Results

23 (46%) patients were males and 27 (54%) were females (Table 1). The mean and median ESR before and after receiving IVIG have been shown in Table 2. The mean of ESR before IVIG was 31.8 ± 29.04 and after IVIG was 47.2 ± 36.9; this difference was meaningful (*P* = 0.05). In male group the mean ESR before IVIG was 34.6 ± 33.5 and after IVIG was 49.1 ± 36.2; the difference with (*P* = 0.003) is statistically significant. Before receiving IVIG the mean ESR was 29.4 ± 25.2 in females and after receiving IVIG it was 45.6 ± 38.04; this difference with (*P* = 0.001) is significant. Results of different age groups, 6 patients less than 28 days, 13 patients from 1 month to 1 year, 20 patients from 1 to 6 years old, and 11 patients from 6 to 12 years, have been shown in Table 3.

## 4. Discussion

ESR is known as an important factor in the evaluation of infectious and inflammatory processes. [2, 4]. Important point for the use of the ESR as an APR is its role in the evaluation of response to treatment; decreasing levels of ESR are considered as a marker of response to therapy.

Plasma fibrinogen and globulins are the major factors affecting ESR [1, 2]. Fibrinogen is the strongest aggregator [2]. And its concentration in the blood is directly related to the rate of ESR [1]. Alpha and gamma globulin provide half the ability of fibrinogen and albumin has the lowest ability in the sediment of red cells.

However, as mentioned, IVIG as an intravenous immune globulin is used in many diseases [6, 7]. Whether the administration of IVIG, influences the ESR value in monitoring of the response to therapy?

In review of the literature generally two studies were found about the effect of IVIG on ESR. One of them has been done on Kawasaki patients and has discussed numerous parameters of protein and also ESR affected by IVIG [10] and the subsequent study has been limited to patients with myasthenia gravis and Guillain-Barré syndrome showing numerous parameters and blood ESR affected by IVIG [11], but this study has discussed 16 different diseases following the administration of IVIG and has focused on the changes in ESR.

IVIG as an immunoglobulin, mostly IgG [7], has aggregator effect on red cells and prevents their negative discharge forces [2]. Although IVIG has many anti-inflammatory effects [7] and it is expected to reduce ESR rate, in vivo its biologic effect is more effective than the immune modulation effect.

The mean ESR was 31.8 ± 29.04 before receiving IVIG and after IVIG it was 47.2 ± 36.9, with significant differences (*P* ≤ 0.05).

These changes were statistically significant in both sexes; it could be interpreted because of lack of physiologic difference in children.

In different age groups except the neonatal period, 1 month to 1 year (13 patients), 1 to 6 years (20 patients), 6 to 12 years (11 patients), the mean ESR before IVIG and after receiving IVIG increased with significant differences; *P* values were, respectively, *P* = 0.001, *P* = 0.025, and *P* = 0.006.

Rapid physiological changes in various neonatal plasma proteins such as albumin and fibrinogen are the variables leading to these results [12]. Different immune regulatory functions of IVIG through its interaction with innate and adaptive immune system and immune homeostasis in neonatal period could be another reason [13].

Among the APR, the ESR is the most valuable criteria for evaluating and monitoring of response to inflammation [1]; on the basis of this study on patients who are receiving IVIG as a therapy, ESR increased falsely (noninflammatory rising); therefore use of ESR for monitoring of response to treatment may not be reliable.

Based on the knowledge of authors and review of the literature this study seems to be the only work about ESR and IVIG administration effect in children. Although these results do not apply to neonatal group we suggest that, in patients who receive IVIG, interpretation of ESR should be used cautiously on the followup process.

## Figures and Tables

**Table 1:** Patients profile.

Patient	Disease	Age	Sex	ESR before IVIG	ESR after IVIG
1	Icter	4 days	M	9	32
2	Icter	2 days	F	7	10
3	Icter	2 days	F	72	61
4	Icter	2 days	M	2	95
5	Icter	10 days	M	24	28
6	Icter	4 days	M	2	2
7	ITP	35 days	F	5	12
8	ITP	2 months	M	42	75
9	Sepsis	50 days	M	6	15
10	ITP	2 months and 5 days	F	47	117
11	ITP	2 months	M	16	56
12	ITP	4 months and 19 days	M	5	8
13	ITP	17 months	M	5	95
14	Sepsis	7.5 months	F	52	65
15	Fever resistant	8 months	F	17	89
16	Kawasaki	10 months	M	54	97
17	Kawasaki	1 year	M	90	112
18	ITP	1 year	F	65	91
19	ITP	1 year	F	12	17
20	GBS	14 months	F	89	120
21	Pneumonia + brain tumor	15 months	F	137	105
22	GBS	1.5 year	M	18	23
23	ITP	1.5 year	M	45	72
24	ITP	2.5 years	F	10	27
25	Epilepsy resistant	2.5 years	M	2	7
26	Epilepsy resistant	2.5 years	M	4	4
27	Bulbar palsy + cyanosis	2 years and 6 months	M	21	71
28	Epilepsy resistant	2 years and 11 months	F	2	5
29	ITP	3 years	F	14	28
30	ITP	3 years	F	6	17
31	Kawasaki	3 years and 4 months	M	115	85
32	Aplastic anemia + fever	4 years	F	77	77
33	GBS	4 years	M	33	62
34	Encephalitis	4 years and 6 months	F	16	33
35	ADEM + fever	5 years	F	3	22
36	Brain atrophy + epilepsy	5 years and 4 months	M	4	18
37	Encephalitis	5 years and 7 months	M	33	33
38	SLE + thrombocytopenia	6 years	F	24	44
39	Steven Johnson	6 years	M	8	13
40	ITP	7 years	F	18	31
41	GBS	7 years	M	43	64
42	Vasculitis	7 years	F	48	79
43	ITP	8 years	F	20	27
44	ITP	8 years	F	23	32
45	Bruton	10 years	M	2	4
46	Bruton	10 years	M	3	2
47	ITP	11 years and 4 months	M	10	15
48	ITP	11 years and 7 months	F	7	21
49	Dawn + pancytopenia	12 years	M	75	121
50	ITP	12 years and 7 months	M	10	23

**Table 2:** Mean and median of ESR.

Patients	Mean ESR		SD		*P*	Median	
	Before	After	Before	After		Before	After
50	31.8	47.2	29.04	36.9	0.05	16.5	32

**Table 3:** Different ages variation.

Age	Number	Before IVIG	After IVIG	*P* value
0-1 month	6	27.03 ± 19.33	38 ± 34.6	*P* = 0.28
1 month–1 year	13	32 ± 27.9	65.3 ± 39.9	*P* = 0.001
1 year–6 years	20	39.8 ± 33	43.3 ± 34.5	*P* = 0.025
6 years–12 years	11	23.5 ± 22.7	38 ± 35.9	*P* = 0.006
